# Interplay Between Fatty Acids, Stearoyl-Co-A Desaturase, Mechanistic Target of Rapamycin, and Yes-Associated Protein/Transcriptional Coactivator With PDZ-Binding Motif in Promoting Hepatocellular Carcinoma

**DOI:** 10.1016/j.gastha.2022.07.017

**Published:** 2022-08-03

**Authors:** Jihane N. Benhammou, Jim Sinnett-Smith, Joseph R. Pisegna, Enrique J. Rozengurt

**Affiliations:** 1 Vatche and Tamar Manoukian Division of Digestive Diseases, David Geffen School of Medicine at the University of California, Los Angeles, California; 2 Division of Gastroenterology, Hepatology and Parenteral Nutrition, Greater Los Angeles Veterans Affairs Healthcare System, Los Angeles, California

**Keywords:** Outcomes, Regulation, Translational oncology

## Abstract

Nonalcoholic fatty liver disease has reached pandemic proportions with one of its most consequential complications being hepatocellular carcinoma (HCC). Nonalcoholic fatty liver disease-related HCC is becoming the leading indication for liver transplantation in the United States. Given the scarcity of available organs, early detection and prevention remain key in prevention and management of the disease. Over the years, the yes-associated protein (YAP)/transcriptional coactivator with PDZ-binding motif (TAZ) pathway emerged as a key signal transduction pathway in the pathogenesis of HCC. In this review, we explore the interplay between the YAP/TAZ pathway as a point of convergence in HCC pathogenesis. We review the evidence of how lipid reprogramming and key lipid pathways, saturated and monounsaturated fatty acids (through the rate-limiting enzyme stearoyl Co-A desaturase), the mevalonic acid pathway (the role of statins), and mechanistic target of rapamycin all play critical roles in intricate and complex networks that tightly regulate the YAP/TAZ pro-oncogenic pathway.

## Introduction

Primary liver cancer is the second leading cause of cancer death worldwide.^1^ Hepatocellular carcinoma (HCC), which comprises 90% of the cases, commonly presents in patients with hepatic fibrosis associated with chronic hepatitis B virus or hepatitis C virus, excessive alcohol consumption, or the metabolic syndrome (MetS). As described below, the genetic landscape of HCC is complex and includes mutations in the Wnt/β-catenin (CTNNB1) pathway, chromatin remodeling, telomere maintenance, and inactivation of p53.

## Nonalcoholic Fatty Liver Disease Associated HCC Burden

In the USA, liver cancer incidence rates have more than tripled since 1980, while the cancer-associated death rates have more than doubled during this time. In parallel, nonalcoholic fatty liver disease (NAFLD), a common condition in patients with obesity and type 2 diabetes, has reached pandemic proportions and continues to rise.^2^ In the era of direct-acting antiviral therapy for hepatitis C virus and improved hepatitis B virus treatment, NAFLD-HCC is predicted to become the leading indication for liver transplantation.^3^ As liver transplantation remains a scarce resource with recent evidence suggesting that HCCs are transplanted less often following changes in United States Organ Procurement Network policy,^4^ understanding its underlying mechanisms is of upmost importance to develop and implement chemoprevention and early detection programs, as well as tailor treatment approaches.

NAFLD and HCC are complex diseases that result from genetic and environmental interactions.^5^ Many clinical potentially modifiable risk factors have been associated with NAFLD-HCC development, including features of the MetS, as defined by the clustering of type 2 diabetes, hypertension, dyslipidemia, and obesity, where each condition plays an additive or synergistic role in the pathogenesis of NAFLD-HCC.^6^^,^^7^ Although statins are commonly prescribed for the treatment of dyslipidemia and the MetS, their use has been shown to be *protective* in all etiologies of HCC,^8^^,^^9^ including in nonalcoholic steatohepatitis (NASH), the more severe form in the NAFLD spectrum.^10^^,^^11^

The mechanisms involved in NAFLD-related HCC pathogenesis are therefore of major fundamental and translational importance but remain incompletely understood. In a large veteran population, the estimated incidence of HCC in the patients with NAFLD is 0.21/1000 person-years (PY), with the highest incidence occurring in the subpopulation of patients with cirrhosis (10.6/1000 PY).^7^ Simon et al^6^ demonstrated similar findings in a European population-based cohort of biopsy-proven NAFLD and NASH, where HCC occurred at an incidence of 6.2/1000 PY for patients with underlying cirrhosis (95% confidence interval 4.2–8.8/1000 PY). The incidence of HCC was lower for patients with biopsy-proven simple steatosis (n = 5939) at 0.8/1000 PY and NASH without fibrosis (n = 1050) at 1.2/1000 PY.^6^ The differences in incidence and disease progression likely reflect the heterogeneity in the NAFLD population, potentially nonmodifiable genetic differences, and their gene-environment interactions. There is evidence from previous genome-wide association studies demonstrating a predominance of NAFLD in Hispanic patients, followed by Caucasians and African Americans,^12^ which have been further substantiated in the HCC population.^13^ Unlike viral and ethanol-associated causes of HCC, NAFLD patients represent a distinct high-risk population where 20%–30% of all HCCs occur in the absence of cirrhosis.^14^^,^^15^ Although current guidance does not recommend HCC screening in patients without cirrhosis, the estimated disease burden globally^16^ and health-care-associated costs^17^ prompt the need for further research in this population, as outlined by recent professional society recommendations.^18^

## Mutations in HCC Development

Heritability of NAFLD and NASH has been suggested based on early observations of the clustering of disease in families and twins.^19^^,^^20^ In NAFLD-related HCC, teasing out inherited genetic contributions from chronic fibrosis/cirrhosis vs NAFLD-specific causes using genome-wide association studies remains a challenge and is still being investigated. Independent of its genetic predisposing factors, HCC often results from an accumulation of somatic mutations that lead to tumor initiation and progression. Studies have demonstrated that each HCC has about 40–60 somatic mutations, several of which converge on key molecular pathways.^21^ Many mutations affect telomere maintenance (telomere reverse transcriptase activation); Wnt activation (mutation in *CTNNB1* which encodes β-catenin, *APC*, and *AXIN1*); the tumor suppressor p53 gene (*TP53*); chromatin remodeling (including in the adenine-thymine-rich interactions-rich interaction domain 1A or *ARID1A*); activation of the PI3K/mechanistic target of rapamycin (mTORC1); and activation of receptor tyrosine kinases.^21^, ^22^, ^23^, ^24^, ^25^ The majority of mutations identified have been shown not to be actionable therapeutically.^22^^,^^26^ Of the multiple pathways identified, certain ones deserve special attention because of their common occurrence. For instance, β-catenin activation through point mutations of *CTNNB1* and mutation of *TP53* occurs in up to 50% of HCC cases.^25^^,^^27^^,^^28^ HCCs can be subclassified into proliferation and nonproliferation classes with some overlaps in the mutational landscape, including in β-catenin activation.^22^^,^^29^ An emerging area of interest is the interplay among lipid metabolism, mTORC1, and the Hippo pathway in HCC. These represent major foci of this article, which are discussed in subsequent sections.

## The Lipid Environment and NAFLD-Related HCC

It is widely accepted that growing cells require unsaturated fatty acids and cholesterol as essential building blocks for biogenesis of cellular membranes. Early on, investigators observed that cancer cells also synthesize lipids.^30^ Lipids, and specifically fatty acids, have been shown to have an integral role in changing the tumor environment (TME) to meet the demands of rapid cell proliferation observed in cancer cells, where oxygen and nutrients are scarce.^31^ This hallmark of cancer cells has been termed “metabolic reprogramming,”^32^ and has been linked to clinical aggressiveness of many tumors.^33^ This is supported by a large body of evidence that suppression of lipogenic pathways decreases cell growth in both *in vitro* and *in vivo* models.^34^^,^^35^ Understanding tumor lipid metabolism and the crosstalk between the lipidome and the pathways that drive HCC has therefore advanced the targeting of lipid pathways in cancer treatments and includes the use of fatty acid synthase (FASN) TVB-2640 for non-small-cell carcinoma (NCT03808558), high-grade sarcoma (NCT03032484), and triple-negative breast cancer (NCT03179904), which are all currently investigated in clinical trials.

The study of the lipidome has only recently been extended to understanding the role of lipids in the pathogenesis of HCC. Most human studies have been conducted in viral etiologies of HCC and have focused on urine and serum lipid analyses with the aim to understand tumor biology and develop clinical biomarkers.^36^, ^37^, ^38^, ^39^, ^40^, ^41^, ^42^ Given that serum and tissue lipids measurements are not always concordant,^43^ other studies have focused on understanding the lipid profile of the HCCs and adjacent nontumor tissue controls.^44^^,^^45^ Few lipidomic studies have been conducted in NAFLD-HCC patients. Lewinska et al^46^ recently studied the serum lipidome profile of patients with NAFLD-HCC (n = 27) and compared it to that of patients with ethanol and viral etiologies of HCC (n = 32), morbidly obese patients (n = 102), and healthy controls (n = 35). Compared to patients with ethanol and viral etiologies of HCC and obsese patients with NAFLD, NAFLD-HCC patients had higher triglycerides (47:0 and 45:1) and phosphatidylcholine (16:0/17:0, 18:2/0:0, 0:0/18:2) levels but lower monounsaturated fatty acids (MUFAs), polyunsaturated fatty acids, and linoleic acid, after adjusting for age, sex, and body mass index.^46^ The authors propose that the lower MUFA and polyunsaturated fatty acids pool may be related to an increase in the uptake of these lipid species by the HCCs from the serum to sustain their growth and proliferation. Consistent with these findings, Muir et al,^47^ identified that stearoyl-Co-A desaturase (SCD), the rate-limiting enzyme in the conversion of saturated fatty acids (SFAs) to MUFAs, which is preferentially found on the endoplasmic reticulum, was upregulated in human NAFLD-related HCC samples and *Pten*-null NASH-HCC murine models. While unsaturated fatty acids are required for cellular proliferation and membrane biogenesis, fatty acids also play important signaling functions that remain much less understood.

## SCD, mTORC1, and HCC

As indicated above, SCD plays a critical role in regulating the ratio of unsaturated fatty acids/SFAs. MUFAs represent the precursors of the main components of cellular membranes and are crucially important in the pathogenesis of the MetS, NAFLD, NASH, and HCC pathogenesis.^48^, ^49^, ^50^ This is supported by clinical studies demonstrating worse overall survival of patients with HCCs who demonstrate a high SCD expression, compared to HCCs with low SCD expression. Consistent with SCD playing a critical role in HCC tumor growth, Bansal et al^51^ demonstrated the inverse relationship between SCD gene expression patterns and HCC tumor differentiation. Similarly, the role of SCD in HCC is further supported by an elevated serum saturated to unsaturated phosphatidylcholine (16:0/16:1) level in patients with cirrhosis and HCC.^50^^,^^52^ Mechanistic studies of SCD and MUFAs in chronic liver disease point to selective effects of SCD on different resident liver cell types including M1 macrophage, hepatic stellate cells, and hepatocytes.^53^ These data suggest that the TME, supported by these other cell types, may play an integral role in HCC growth and aggressiveness. However, detailed human cell-type studies are lacking and have only recently been able to be identified with the advent of single-cell and single-nucleus RNA sequencing.^54^, ^55^

**Figure 1:**
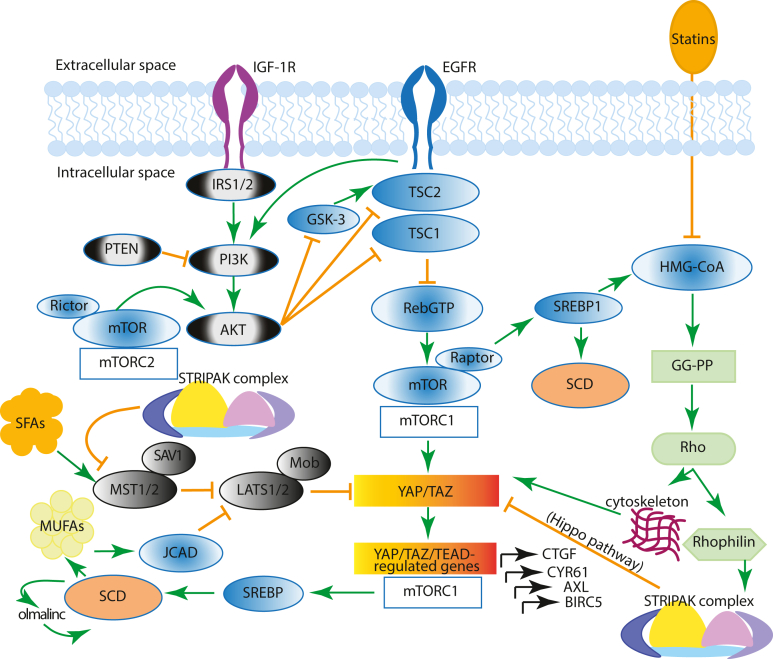
Proposed model for cross-talk of different pathways converging on the YAP/TAZ pathway in HCC pathogenesis.

**Figure 2:**
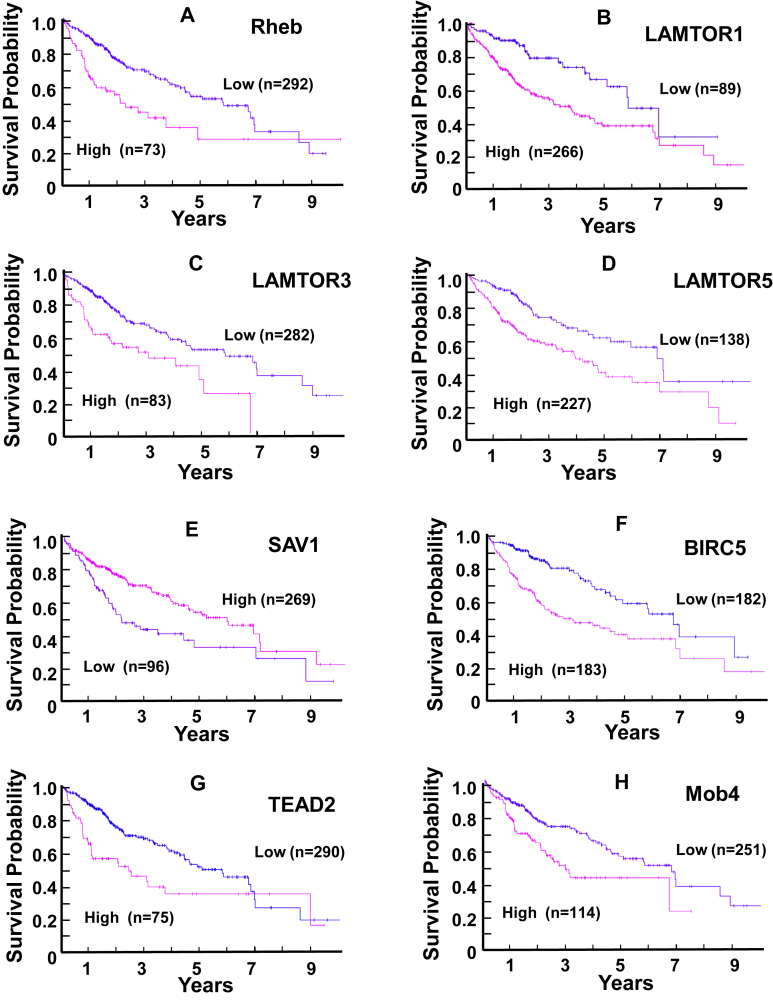
Kaplan-Meier plots for gene expression of the signaling network in HCC. Images were reproduced from the Human Protein Atlas (version 17) available from www.proteinatlas.org.^59^ Transcriptomics data were available from 365 patients in total with 119 female and 246 male patients. A majority of patients (n = 235) were still alive at the time of data collection. The stage distribution was (i) 170 patients, stage (ii) 84 patients, stage (iii) 83 patients, stage (iv) 4 patients, and 24 patients with missing data information. The links to the specific genes shown are as follows: (A) Rheb: https://www.proteinatlas.org/ENSG00000106615-RHEB/pathology/liver+cancer/ (B) LAMTOR1: https://www.proteinatlas.org/ENSG00000149357-LAMTOR1/pathology/liver+cancer (C) LAMTOR3: https://www.proteinatlas.org/ENSG00000109270-LAMTOR3/pathology/liver+cancer (D) LAMBOR5: https://www.proteinatlas.org/ENSG00000134248-LAMTOR5/pathology/liver+cancer (E) SAV1: https://www.proteinatlas.org/ENSG00000151748-SAV1/pathology/liver+cancer (F) BIRC5: https://www.proteinatlas.org/ENSG00000089685-BIRC5/pathology/liver+cancer (G) TEAD2: https://www.proteinatlas.org/ENSG00000074219-TEAD2/pathology/liver+cancer (H) Mob4: https://www.proteinatlas.org/ENSG00000115540-MOB4/pathology/liver+cancer.

As shown in Figure 1, SCD expression is regulated at multiple levels,^56^ including by the sterol regulatory element-binding protein 1 (SREBP1), the master transcription regulator of lipid biosynthesis, which has higher expression in HCC through mammalian target of rapamycin (mTOR) signaling. mTOR Functions as a catalytic subunit in 2 distinct multiprotein complexes, the mTORC1, characterized by the subunit Raptor (regulatory-associated protein of mTORC1), and mTORC2, characterized by the subunit Rictor (Raptor-independent companion of mTORC2). The heterodimer of the tumor suppressor TSC1 and TSC2 represses mTORC1 activity by acting as the GTPase-activating protein for Rheb, a potent activator of mTORC1 in its guanosine triphosphate-bound state (Figure 1). Using an interactive open-access database (www.proteinatlas.org/pathology), we found that higher expression of Rheb is significantly associated with poor survival in HCC cases (Figure 2A). In the presence of amino acids, activated Akt and/or an extracellular signal-regulated kinasesK/p90RSK phosphorylate and uncouple TSC1/TSC2 from Rheb, leading to Rheb-GTP accumulation and mTORC1 activation at lysosomal membranes, which then promotes cell growth.^57^ Ragulator complex proteins LAMTOR 1, 3, and 5, which are involved in amino acid sensing and mTORC1 activation, are also significantly associated with unfavorable prognosis (survival) in HCC (Figure 2B–D). The association of Rheb and LAMTORS 1, 3, and 5 with unfavorable prognosis emphasizes the importance of mTORC1 function in HCC development. In turn, mTORC1 increases lipogenesis through regulation of SREBP1 at several levels, including trafficking, processing, and transcription^58^ that culminate in its nuclear localization and transcriptional activation. In turn, nuclear SREBP1 induces the expression of lipogenesis genes, including *SCD*.

## SCD and MUFAS in the Control of the Hippo Pathway

In recent years, the ratio of SFA to MUFAs has been implicated in the regulation of the highly conserved Hippo tumor-suppressor pathway and in the function of its downstream effectors in several cell types. The transcriptional coactivators yes-associated protein (YAP) and its paralog WW-domain-containing transcriptional coactivator with PDZ-binding motif (TAZ) are critical effectors of the Hippo pathway and fundamental points of convergence and intersection of other signal transduction pathways implicated in the regulation of development, metabolism, organ size, positional sensing, tissue regeneration, and tumorigenesis.^60^ The canonical Hippo pathway is a serine/threonine kinase cascade wherein Mst1/2 kinases, in complex with Sav1, phosphorylate and activate Lats1/2, in complex with its regulatory protein MOB1/2 (Figure 1). In turn, LATS1/2 phosphorylate YAP and TAZ. Structurally, YAP and TAZ share nearly half of the overall amino acid sequence and have very similar topology and highly conserved residues located within a consensus sequence phosphorylated by Lats1/2 (HXRXXS). The phosphorylation of YAP by Lats1/2 at Ser-127 and Ser-397 (and equivalent residues in TAZ) restricts its cellular localization to the cytoplasm and reduces its protein stability. When the Hippo pathway is not functional, YAP and TAZ are dephosphorylated and translocate to the nucleus where they bind and activate a number of transcription factors, primarily the TEA-domain DNA-binding transcription factors (TEAD1–4). When the Hippo pathway is active, LATS1/2 phosphorylates YAP and TAZ at multiple sites, thereby restricting their cellular localization to the cytoplasm, thus preventing their interaction with TEADs. An increased expression of SAV1, a protein necessary for the function of Mst1/2 upstream in the Hippo tumor-suppressive pathway, is associated with favorable prognosis in HCC (Figure 2E), whereas, reciprocally, the expression of BIRC5 and TEAD2 has been associated with poor overall survival in HCC (Figure 2F and G). BIRC5, which encodes survivin, is a potent inhibitor of apoptosis and a downstream target of YAP.^61^ In addition, there is emerging evidence that the Mobs are a family of proteins with positive and negative effects on the Hippo pathway.^62^ Accordingly, high expression of Mob 4, a component of the multisubunit protein complex striatin-interacting phosphatase and kinase (STRIPAK) that negatively regulates the Hippo pathway,^62^^,^^63^ is associated with unfavorable prognosis in HCC (Figure 2H).

In line with the associations identified above, several studies in preclinical models indicate that YAP/TAZ play a major role in promoting HCC. An early study demonstrated that YAP overexpression in the liver induces rapid HCC development, implying a potent oncogenic role of this protein.^64^ Further studies using liver-specific knockout of Mst1/2 or Sav1 demonstrated that the Hippo signaling pathway is a critical regulator of mammalian liver growth and a potent suppressor of liver tumor formation.^65^ Reciprocally, heterozygous knockout of YAP in mouse models suppressed the development of HCC-like tumors.^66^ Subsequent multiple studies substantiated that the tumor-suppressive Hippo pathway plays a critical role in liver regeneration^67^ and drug resistance.^68^^,^^69^ Furthermore, YAP promotes mTORC1 activation via downregulation of phosphatase and tensin homolog and increase in amino acid uptake.^70^ In turn, mTORC1 leads to YAP/TAZ accumulation through inhibition of GSK3β,^71^ thus establishing a positive feedback loop (Figure 1). A fundamental crosstalk of the Hippo-YAP pathway with multiple metabolic pathways has been reviewed recently.^72^ The Hippo YAP/TAZ signaling pathway has also been implicated in changing the TME in pancreatic adenocarcinoma, including in macrophages, highlighting the multifaceted role and complex interplay of the Hippo-YAP pathway between cells in cancer development and progression.^73^

Emerging evidence supports the importance of lipid regulation of the Hippo pathway. In endothelial cells, palmitic acid, the most common of the SFA, induces expression of the Hippo pathway kinase Mst1 leading to YAP phosphorylation and nuclear export, therefore inhibiting nuclear YAP function.^74^ Reciprocally, in primary human lung adenocarcinoma cells, knockdown or pharmacological inhibition of SCD impairs the function of YAP and TAZ, implying a positive role of MUFAs in YAP/TAZ activation.^75^ Despite these advances, the mechanisms of YAP/TAZ in HCC remain poorly understood, especially in response to lipid dysregulation and their effects on the tumor microenvironment in different resident cell types of the liver.

## Palmitoylation of TEADs Transcription Factors

Palmitic acid is a SFA with important properties in the MetS,^76^ including in NAFLD and NASH,^77^ that regulates expression of Hippo kinases (see "SCD and MUFAS in the Control of the Hippo Pathway"). Many proteins have been shown to undergo posttranslational modification by S-palmitoylation, including those involved in membrane trafficking and cellular localization.^78^^,^^79^ Using a series of biochemical and genetic approaches, Chan et al^80^ demonstrate that TEADs are autopalmitoylated at conserved cysteine residues, which in turn indirectly regulate YAP and TAZ by changing the conformation of TEAD proteins and, therefore, affecting their downstream association with these coactivators. It was subsequently shown that S-palmityoylation is key in the homeostasis of TEAD protein levels in cells, thus providing another layer of regulation of the Hippo pathway.^81^ FASN is key in the synthesis of plalmitoyl-CoA. Thus, it is conceivable that the FASN benefits observed in early clinical trials for non-small-cell carcinoma, breast cancer, and sarcoma (see “The Lipid Environment and NAFLD-Related HCC”) may be partially due to inhibition of this autophosphorylation loop, which in turn dampens the YAP/TAZ oncogenic pathway. Although this concept is starting to be investigated in HCC *in vitro* cell culture models,^82^ it remains an understudied area of HCC development of progression, especially in the context of NAFLD and NASH where intrahepatic lipid content is high. TEAD palmitoylation is a topic attracting interest as inhibitors of this critical modification are being developed^81^^,^^83^ that could offer novel therapeutic approaches in HCC and other cancers.

## YAP, TAZ, and HCC

Although YAP has been investigated and linked to many malignancies, its role in HCC pathogenesis is only emerging.^84^, ^85^, ^86^, ^87^, ^88^ In a predominant viral hepatitis cohort, patients with high YAP expression in HCCs (n = 177) were more likely to have poorly differentiated HCCs, which was associated with >2-fold increase in HCC-specific death.^89^ Consistent with the mechanism of action of YAP, most tumors (62% had YAP expression) showed nuclear YAP localization.^89^ A recent study uncovered that YAP and TAZ have overlapping and distinct roles in the development of liver carcinogenesis.^90^ Several signaling pathways interact and cross-talk in cancer development to sustain tumor growth, including with YAP. Molecular subclassifications of HCC have been described.^21^^,^^29^ One commonly mutated pathway that has been shown to converge on the YAP/TAZ pathway is the Wnt-β-catenin signaling pathway which affects 30%–50% of all cases.^21^^,^^50^^,^^86^ Mechanistic studies linking YAP/TAZ to Wnt-β-catenin signaling pathway suggest that this regulation occurs, at least in part, indirectly^86^ through SCD and the MUFA/SFA intracellular pool.^50^Lai et al^50^ elegantly demonstrate this in *in vivo* and *in vitro* models where SCD was overexpressed both in activated hepatic stellate cells and hepatocytes. The Wnt-β-catenin in turn increased SREBP1, which stabilized LRP5 and LRP6 via MUFA intracellular pools and promoted liver fibrosis and HCC development.^50^ These data further support TME cross-talk with hepatocytes for HCC pathogenesis.

Although several studies have focused on the role of YAP in HCC, TAZ may play a more important role in the transition from steatosis to NASH and fibrosis, which are precursors to HCC development. TAZ protein was found to be higher in human liver samples and NASH murine models (methionine/choline deficiency or fructose, palmitate, cholesterol) than in controls, with its silencing leading to reversal of inflammation, cell death, and even fibrosis (through stellate cell activation).^91^ The crosstalk between these pathways and convergence on the YAP pathway need further exploration.

## Mevalonate Pathway in the Control of YAP/TAZ

*Statins* are among the most widely prescribed medications in the world to treat dyslipidemia. Although most research on the effects of statins has been in the context of cardiovascular or metabolic diseases, recent large epidemiological studies, including in veterans, indicate a protective effect of statins administration in HCC^92^^,^^93^ and other malignancies.^94^ Specifically, lipophilic statins are associated with a decrease in the incidence of HCC and its associated mortality, compared to hydrophilic statins and statin nonusers.^9^ Although prospective and randomized control trials are ongoing to assess their clinical utility, the mechanisms involved remain poorly understood. Statins are specific inhibitors of the 3-hydroxy-methylglutaryl CoA reductase, the rate-limiting enzyme in the generation of mevalonate, the first step in the biosynthesis of isoprenoids, leading to farnesyl pyrophosphate, geranylgeranyl pyrophosphate, and cholesterol. The transfer of the geranylgeranyl moiety to a COOH-terminal cysteine of Rho GTPases is critical for their function in signal transduction. Indeed, as depicted in Figure 1, active Rho (ie, Rho-GTP) is essential for YAP/TAZ activation, at least in part through actin remodeling^70^ and modulation of the STRIPAK multisubunit complex.^62^ A recent study found that the endoplasmic reticulum protein Nogo-B is highly expressed in both murine and human NAFLD-associated HCCs and leads to lysophosphatidic acid-enhanced YAP oncogenic activity.^95^ Lysophosphatidic acid is known to stimulate YAP via G protein-coupled receptors that promote Rho activation.^96^ Another study concluded that YAP directly interacts with SREBP1c and SREBP2 on the promoters of the FASN and 3-hydroxy-methylglutaryl CoA reductase, thereby stimulating their transcription and promoting hepatocyte lipogenesis and cholesterol synthesis.^97^ Several studies linked mevalonate metabolism and YAP/TAZ activity and identified lipophilic statins as YAP/TAZ inhibitors in a variety of cell types,^98^, ^99^, ^100^ but the impact of statins on YAP localization, phosphorylation, and activity in HCC cells remains unknown. The possibility that statins impair actin remodeling and/or STRIPAK activity in response to multiple agonists and growth factors that induce Rho activation (ie, conversion of Rho-GDP to Rho-GTP) makes molecular mechanisms intriguing in the context of HCC and other cancers but remains untested. Interestingly, it has been identified that the ATP-binding cassette A1 transporter is a target gene of p53.^101^ Activation of the ATP-binding cassette A1 transporter results in reverse cholesterol transport, which leads to decreased SREBP2, the master transcription factor of cholesterol biosynthesis, which in turn leads to repression of the mevalonic acid pathway.^101^

## Conclusion

Treatment of advanced and unresectable HCC has greatly changed over the recent years with the advent of immunotherapy and advances in locoregional therapies.^102^ For decades, sorafenib, a multiprotein kinase inhibitor, was the first-line treatment for advanced HCC. Its long-standing use informed patterns of resistance and how it affected HCC recurrence. In the context of this discussion, it is of interest that several recent studies identified YAP as a major mediator of HCC resistance to sorafenib.^103^^,^^104^ Similarly, Hippo signaling has been recently been shown to be a target of regorafenib, which has a similar mechanism of action to sorafenib, resistance in HCC using a CRISPR-Cas9 screen.^68^

In the light of the signaling network discussed herein, the exciting possibility of synergism between statins, SCD inhibitors, mTORC1 inhibitors (including rapamycin and its analogs), and kinase inhibitors in dampening HCC cell proliferation and migration and preventing drug resistance through inhibition of YAP/TAZ is a novel therapeutic approach that has not been examined in HCC. Additionally, the use of lipid modulators including statins as chemoprevention methods for NAFLD-related HCC should be further explored to tackle the large health-care and economic burdens anticipated in the near future. Finally, understanding the HCC subclassifications through these different pathways may help explain the heterogeneity of HCC development and treatment responses and would advance the role of precision oncology, especially in NAFLD-HCC, which has distinct features compared to its viral counterparts.
